# Hippo Pathway in Regulating Drug Resistance of Glioblastoma

**DOI:** 10.3390/ijms222413431

**Published:** 2021-12-14

**Authors:** Giacomo Casati, Laura Giunti, Anna Lisa Iorio, Arianna Marturano, Luisa Galli, Iacopo Sardi

**Affiliations:** 1 Neuro-Oncology Unit, Department of Pediatric Oncology, Meyer Children’s Hospital, 50139 Florence, Italy; laura.giunti@meyer.it (L.G.); annalisa.iorio@meyer.it (A.L.I.); arianna.marturano@unifi.it (A.M.); iacopo.sardi@meyer.it (I.S.); 2 Infectious Disease Unit, Department of Health Sciences, University of Florence, 50139 Florence, Italy; luisa.galli@unifi.it

**Keywords:** glioblastoma (GBM), signaling pathways, tumor heterogeneity, tumor microenvironment (TME), Hippo pathway, chemoresistance, immunotherapy

## Abstract

Glioblastoma (GBM) represents the most common and malignant tumor of the Central Nervous System (CNS), affecting both children and adults. GBM is one of the deadliest tumor types and it shows a strong multidrug resistance (MDR) and an immunosuppressive microenvironment which remain a great challenge to therapy. Due to the high recurrence of GBM after treatment, the understanding of the chemoresistance phenomenon and how to stimulate the antitumor immune response in this pathology is crucial. The deregulation of the Hippo pathway is involved in tumor genesis, chemoresistance and immunosuppressive nature of GBM. This pathway is an evolutionarily conserved signaling pathway with a kinase cascade core, which controls the translocation of YAP (Yes-Associated Protein)/TAZ (Transcriptional Co-activator with PDZ-binding Motif) into the nucleus, leading to regulation of organ size and growth. With this review, we want to highlight how chemoresistance and tumor immunosuppression work in GBM and how the Hippo pathway has a key role in them. We linger on the role of the Hippo pathway evaluating the effect of its de-regulation among different human cancers. Moreover, we consider how different pathways are cross-linked with the Hippo signaling in GBM genesis and the hypothetical mechanisms responsible for the Hippo pathway activation in GBM. Furthermore, we describe various drugs targeting the Hippo pathway. In conclusion, all the evidence described largely support a strong involvement of the Hippo pathway in gliomas progression, in the activation of chemoresistance mechanisms and in the development of an immunosuppressive microenvironment. Therefore, this pathway is a promising target for the treatment of high grade gliomas and in particular of GBM.

## 1. Glioblastoma Chemoresistance

Among the different malignant gliomas, glioblastoma (GBM), which accounts for about 60–70% of all gliomas, is classified as a World Health Organization (WHO) grade IV tumor based on histopathological features, and it represents the most frequent and malignant tumor of the Central Nervous System (CNS), affecting both children and adults with a slight predominance in males.

Despite experimental investigation in this field and the improved therapeutic strategies, GBM remains essentially incurable, with an overall survival time ranging from 12 to 18 months, as less than 5% of patients survive longer than five years after diagnosis. Currently, consolidated first line treatment options for human GBM are radiotherapy and chemotherapy with Temozolomide (TMZ). One hope for a better clinical outcome is to identify targets that play essential roles in mediating the microenvironment-derived survival signal, drug resistance or sensitize the response of GBM cells to radiation and chemotherapeutic drugs. Multidrug resistance (MDR) remains a great challenge to GBM therapy. This cellular phenomenon is the main cause of disease relapse, normal tissue infiltration and distant metastasis.

MDR is due to various and complex mechanisms including crosstalk between tumor microenvironment (TME) and GBM stem cells (GSCs), deregulated signaling pathways, abnormal expression of a specific protein, and cell-to-cell communication mechanisms [1] (Table 1).

The deregulation of the Hippo pathway represents a mechanism that causes MDR in GBM cell lines. In particular, the overexpression of TAZ (Transcriptional Co-activator with PDZ-binding Motif) decreases the cytotoxic effect of TMZ by upregulating the MCL-1 protein and thus making the U87MG and U251 cell lines apoptosis resistant [2].

Moreover, the overexpression of the YAP-TAZ-TEAD complex provokes GBM cell resistance to TMZ treatment by up-regulation of the Hippo pathway downstream target genes. In detail, CTGF (connective tissue growth factor) and Cyr61 (cysteine rich angiogenic inducer 61) genes are upregulated through TGF-β1-dependent activation of Smad/ERK signaling [3].

Moreover, another issue of GBM chemoresistance is due to CD109 protein. CD109 binds to the GP130 receptor and promotes the activation of the IL-6/STAT3 signaling by increasing GSCs stemness and tumorigenicity, and MDR. CD109 can also activate the Hippo pathway in response to damage by conferring radioresistance and chemoresistance through the upregulation of its target genes. Interestingly, the loss of CD109 in vivo leads to a reduction in nuclear YAP (Yes-Associated Protein) level, STAT3 activation and GSCs stemness and therefore reduces tumorigenicity [4,5].

In addition to the intrinsic tumor chemoresistance, drugs delivery through the blood brain barrier (BBB) is very complicated due to abnormal/de novo expression of specific drugs transporters in cancer cells and/or in the endothelial cells forming the BBB [6,7]. Therefore, GBM is characterized by an overexpression of specific drug efflux pumps called ATP-binding cassette (ABC) superfamily such as P-gp, breast cancer resistance protein (BCRP/ABCG2) and MRP1 [8,9]. Furthermore, multiple genetic mechanisms appear to be involved in the resistance phenotype of GBM. GSCs are characterized by altered DNA repair mechanisms, as well DNA damage response (DDR) and mismatch repair (MMR), physiologically involved in the maintenance of genetic stability [10,11,12].

DDR contributes to remove DNA lesions caused by conventional DNA-damaging agent used for GBM such as TMZ and ionizing radiation (IR) conferring chemoresistance phenotype to GBM cells [13]. Failures in MMR are associated with glioma cells TMZ resistance. Repeated exposures to TMZ can induce acquired *MSH6* mutations in GBM cells turning off the MSH2/MSH6 dimer and prompting cytotoxicity [14,15].

When the Hippo pathway is “on”, it exploits the DNA repair mechanisms both in immortalized cell lines and in primary cells from GBM patients [16,17]. An intriguing study demonstrated that YAP plays a radioresistant role on gliomas by repairing DNA damage.

After radiation, YAP is able to promote the expression of the FGF2 factor and activate the MAPK-ERK pathway. The YAP-FGF2-MAPK signaling is a key mechanism of radioresistance in GBM [18].

Another study, through a microarray analysis, evaluated the variation of the gene expression profile after proton irradiation. The data show that the Hippo pathway is one of the highly deregulated signaling after proton-therapy irradiation. The Wnt pathway is also deregulated after irradiation. Many authors have already proved that these two molecular signaling interact with each other; the main novelty is that YAP is capable of activating the Wnt/β-catenin pathway, which in turn promotes tumor growth and resistance to radiation in GBM cell lines [19,20,21,22].

The cross-talk between mTOR and Hippo pathway is further evidence of chemoresistance. Indeed, mTORC2 subunit is able to phosphorylate YAP on serine 436 (Ser436) allowing the activation of the Hippo signaling independently from the canonical pathway. This aberrant interplay promotes growth, migration, and drug resistance in both cell lines and GBM patient samples [23,24,25].

About 30–60% of GBM presents methylation of MGMT promoter associated with an increased sensitivity to TMZ and prolonged survival [26,27]. The lack of methylation causes a different correction of the DNA lesion induced by TMZ, generating an incorrect mispairing and consequently an anomalous activation of MMR system. The abnormal loop mode activation of MMR, called “futile cycle”, can provoke the induction of DNA double strand breaks (DSBs) and the activation of specific signaling pathways regarding cellular cycle arrest and cell death. Another molecular mechanism that plays an important role in the genesis and development of chemoresistance in GBM is the aberrant expression of microRNA (miRNAs) [28,29,30,31,32]. miRNAs are short non-coding RNAs molecules that control the expression of genes involved in different cellular processes (proliferation, apoptosis, cell differentiation, anti-viral defense), and their aberrant expression has been reported in tumors [33,34,35,36].

GBM miRNA targets are drug transporter genes, proteins involved in ABCB1/P-gp-mediated chemoresistance and genes involved in DNA repair mechanisms [37,38,39,40,41,42,43,44]. Recent studies, most regarding the release of exosomes, show new interesting data regarding cell-to-cell communication.

Exosomes are cell–cell communication extracellular vesicles with a heterogeneous content of molecules such as protein (receptor, enzymes, and transcription factors), nucleic acids (DNA, mRNA, miRNA, long non-coding RNA (lncRNAs)), growth factors, and lipids [45]. Exosomes participate in both physiological (coagulation and immunosurveillance) and pathological processes (chemoresistance and carcinogenesis) [46,47,48].

Chemoresistance induced by the release of exosomes can involve many cellular pathways such as a TME modulation that induces the epithelium–mesenchymal transition process (EMT). Alternatively, exosome release may activate miRNA-mediated gene expression regulatory mechanisms; furthermore, it may also promote immune escape, angiogenesis and metastasis [49]. Moreover, chemotherapeutic agents can be internalized in exosomes and therefore excluded from drug-resistant tumor cells improving drug efficacy [50]. The delivery of exosomal cargo, which contains drug efflux pumps, fusion genes and lncRNAs, to cancerous cells is associated with drug resistance in GBM [51,52,53,54,55,56] (Table 2).

Another mechanism that contributes to chemoresistance is the inhibition of chemotherapy-induced apoptosis by the tumor. In this regard, the Notch signaling is known to modulate apoptosis in cancer. Specifically, blocking Notch pathway causes the induction of apoptosis. In GBM, inhibition of Notch signaling induces apoptosis in TMZ and Etoposide resistant cells [57,58,59].

However, the Hippo pathway is capable of activating the Notch signaling by the upregulation of JAG-1 protein, thus decreasing apoptosis induced by chemotherapy and increasing chemoresistance [60].

## 2. Immunosuppressive Mechanisms in Glioblastoma

Recurrence is a classical GBM hallmark that prevents good prognosis. Currently, second-line therapy has not been developed for all patients (about 50% of patients did not get any therapy during the progression) [61,62]. Many studies show that GBM is characterized by an immunosuppressive microenvironment due to a rise of factors released by tumor cells such as programmed cell death protein-1 (PD-1), indolamine 2, 3dioxygenase (IDO), STAT3 and FASL. Moreover, microglia cells can produce TGF-B and IL-1, which, in turn, promote systemic immunosuppression and control local myeloid and lymphatic immune cells [63].

Myeloid cells alter the expression of various extracellular and intracellular mediators; they ensure an immunosuppressive microenvironment and therefore favor the tumor [64]. All of these factors change the phenotype of cytotoxic T lymphocytes (CTLs), enhancing the levels of immunosuppressive markers such as PD-1.

Many studies take advantage of these concepts and focus on promoting antitumor immune responses. For instance, anti-PD-1 and anti-CTLA-4 treatments or vaccine therapies are performed to destroy tumor cells containing GBM-associated antigens such as EGFRvIII [65].

Differently, viral oncolytic therapy is a treatment that involves the application of a virus able to activate the tumor immune system. Oncolytic viruses are attenuated and are spread into tumor cells by exploiting the lack of a viral defense mechanism [66].

Another experimental approach to stimulate the antitumor immune response can be performed using CAR T lymphocytes (chimeric antigen receptor T cells modified), although they cause inflammation, increased intracranial pressure and CNS neurotoxicity. Therefore, this therapeutic strategy is very limited and complicated [67,68,69].

In addition to the factors mentioned above (PD-1, indolamine 2, IDO, STAT3, FASL, TGF-B and IL-1), TME contain high amounts of tumor associated macrophages (TAMs); they are highly infiltrating and present in two different phenotypes, M1 and M2. In particular, M1 TAMs perform anti-tumor functions, conversely, M2 TAMs are induced by IL-4, IL-13 and glucocorticoids with tumorigenic functions. TME consists mostly of M2 TAMs; however, the precise mechanism underlying the polarization of TAMs remains to be elucidated [70]. TAMs are recruited by various cytokines and chemokines secreted by cancer cells, such as monocyte chemotactic protein 1 (Mcp-1) and colony stimulating factor-1 (CSF-1) and are often the main cause of poor prognosis in multiple types of tumor such as colon cancer [71,72,73,74].

Resistance to immunotherapies is due to the low immunogenicity of GBM and numerous immunosuppressive stressors in the microenvironment [75].

The Hippo pathway is one of the most studied molecular mechanisms for the regulation of tumor proliferation, migration, angiogenesis and invasion in recent years. Several studies show that YAP is able to create communication between the tumor and the immune cells, in particular with the TAMs [70]. In fact, the presence of YAP into the nucleus allows to recruit and activate different inflammatory cytokines for instance IL-6, which modulate the tumor immune response and the tumoral growth. Furthermore, TAMs present in gliomas produce and release IL-6, which can increase the formation of glioma stem cells and induce the accumulation of TAMs in a feed-forward cycle.

**Figure 1:**
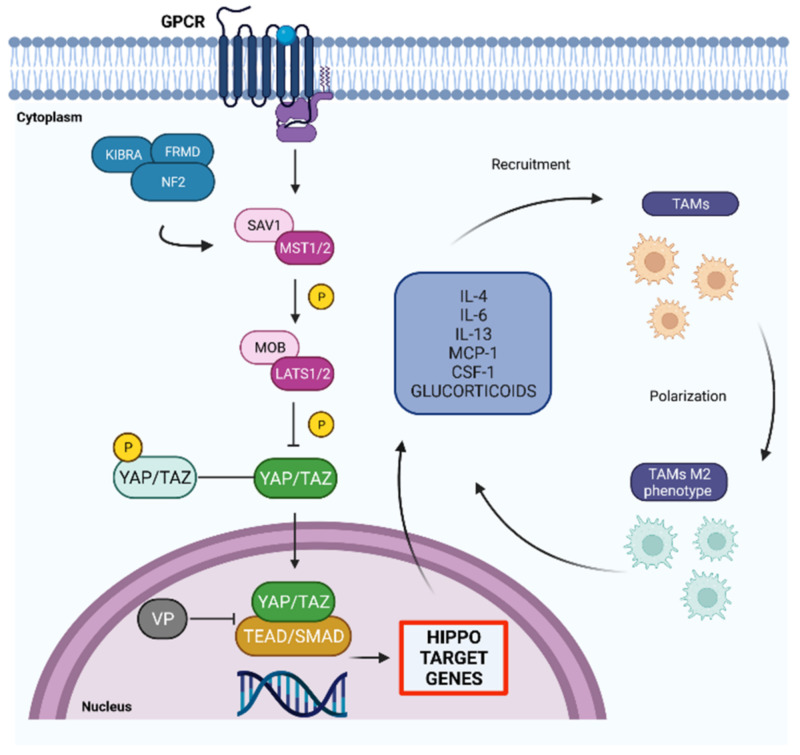
TAMs recruitment and polarization from M1 to M2 are triggered by the Hippo pathway. Nuclear localization of YAP/TAZ promote the activation of a large amount of inflammatory cytokines which attract TAMs. The M2 phenotype polarization cause migration, proliferation and favor TAMs recruitment. Created with Biorender.com.

Hepatocellular carcinoma cells after treatment with Verteporfin (VP), inhibitor of the YAP-TEAD complex, clearly show a dose-dependent reduction in TAMs recruitment and therefore a lower expression of IL-6 [76,77]. An increased expression of YAP, observed in colorectal cancer studies, is associated with the polarization of TAMs from the M1 to M2 phenotype and with tumorigenesis; conversely, the inhibition of YAP causes a decrease of the cytokines IL-4 and IL -13 leading a return to the M1 phenotype [78] (Figure 1).

YAP is also able to regulate PD-1/PD-L1 (programmed death-ligand 1) expression in various tumors, since modifying TME immunosuppression. For example, BRAF inhibitor-resistant melanoma cells (BRAFi), that aberrantly express YAP, evade the immune response of CD8+ T cells in a PD-L1-dependent manner. The interaction between YAP and PD-L1 is further confirmed in vivo in 472 human melanoma tumor tissues [79]. The abnormal activation of the Hippo pathway in various tumors and therefore the presence of YAP into the nucleus regulates the expression of PD-1/PD-L1, inhibiting antitumor immunity mediated by T cells [80].

Although satisfactory results are not yet obtained, the combination of standard treatment (radiotherapy followed by chemotherapy) with different types of immunotherapeutic approaches (vaccines, CAR T lymphocytes or viral oncolytic therapy) could in the future become part of the standard of care for patients with GBM [81]. Furthermore, numerous studies show an involvement of the Hippo pathway in the regulation of the TME composition, which in turn is able to influence the tumor immune response.

Therefore, studying this molecular pathway is particularly interesting because the development of Hippo pathway molecular target therapies can reduce both the chemoresistance and the immunosuppressive nature of GBM.

## 3. Pathways Involved in Glioblastoma Genesis

The GBM development is characterized by many mutations among different key signaling pathways, including the receptor tyrosine kinase (RKT) ones [82,83], such as the phosphoinositide 3-kinase (PI3K)/protein kinase B (AKT)/mTOR pathway and the Ras/MAPK/ERK pathway, which are involved in the regulation of cell proliferation, survival, differentiation and angiogenesis. The main tyrosine kinase receptor EGFR (epithelial growth factor receptor) mutation is the EGFR variant III. This alteration maintains the receptor into a constitutionally active ligand-independent form, leading to cell proliferation and survival [84]. EGFR-amplified/mutant human GBMs express a high amount of YAP and VP, an inhibitor of the YAP-TEAD complex, is capable to induce apoptosis in patient-derived EGFR- /mutant GBM because it can suppress expression of YAP/TAZ transcriptional targets, including EGFR. YAP/TAZ-TEAD directly regulates transcription of EGFR itself to create a feedforward loop to drive survival and proliferation of human GBM [85].

EGFR signal transduction also stimulates the Ras/MAPK/ERK pathway resulting in migration and cellular proliferation [86]. It is proven a correlation between receptor tyrosine kinase (RTK) signaling and the Hippo Pathway. Indeed, RTK/RAS driven carcinomas, characterized by chemoresistance, metastasis and tumor invasion, show a dysregulation of the YAP-TEAD complex belonging to the Hippo Pathway. TEAD factor is identified as a migration driver both in vitro and in vivo and as a direct transcriptional target of EGFR. Treatment with VP, not only inhibits cell growth and migration but also causes a dose-dependent downregulation of EGFR activity and ERK phosphorylation [87]. EGFR signal transduction drives the recruitment of PI3K to cell membrane with consequent formation of PIP3 (PI-3-phosphate). PIP3 activates downstream molecules like AKT and mTOR [88]. mTOR and the Hippo Pathway coordinately control cell growth and proliferation. The dysregulation of these signaling plays a critical part in gliomagenesis. It is established a cross-talk between these mechanisms; recent studies consider the Angiomotin protein family as a powerful repressor of YAP [89,90]. In particular, the AMOTL2 protein (angiomotin like-2) is identified as a substrate of the mTORC2 subunit. Indeed in GBM cells, AMOTL2 is phosphorylated at the level of serine 760 by mTORC2. AMOTL2 mutation, that mimics the constitutive phosphorylation of Ser^760^, stops its ability to bind and suppresses YAP causing a nuclear increase and therefore a greater expression of its oncogenic targets.

Conversely, AMOTL2 overexpression inhibits YAP-induced transcription *in vitro* [24]. Mutations in the retinoblastoma (RB) pathway are also found in 78% of GBM [91]. RB suppresses cell cycle entry and progression, interacting with the transcription factor E2F [92,93,94]. Other genetic alterations in GBM are at the expense of p53 pathway (altered in 87% of GBM) [91] which is involved in the activation of genes implicated in cell arrest and apoptosis [95]. Hippo Pathway and wild-type p53 cooperate, at multiple levels, as tumor suppressors to cause senescence and apoptosis in response to stressful conditions [96]. Recent in vitro studies show that, in presence of DNA lesions, YAP can interact with p73 (a member of the p53 family) through an independent mechanism of the canonical pathway, inducing apoptosis and reduced proliferation [97,98,99]. In the presence of a mutation, p53 (mtp53) performs an oncogenic activity enhanced by intermediate factors that affect the Hippo pathway. In GBM cells, mtp53 improves PI3K/AKT-mediated phosphorylation of the interacting protein WASP (WIP), a protein associated with the actin cytoskeleton, which promotes YAP stability and cancer stem cell survival [100]. Therefore, Hippo signaling is in the spotlight due to its meaningful roles in both developmental and cancer biology, but it is not the only pathway involved in cell growth and proliferation. These mechanisms are also controlled by other well-known signaling pathways, such as Wnt/β-catenin and TGFβ signaling [101,102].

Indeed, the PI3K/AKT/mTOR pathway is also activated by the Transforming growth factor beta (TGF-β) binding its receptor and in normal conditions it acts as tumor suppressor, inhibiting cell proliferation [103]. Its dysregulation contributes to the GBM pathogenesis, as mutations on TGF-β signaling lead to inflammation, invasion, metastasis, angiogenesis and immune escape [104]. It is demonstrated that various upstream regulators, such as cell polarity, adhesion proteins control Hippo signaling; furthermore, this pathway interacts with other signaling as well Wnt/β-catenin, Notch and MAPK pathways [105]. The role of Wnt pathway in many tumors development, such as in gliomas is well established by several data.

**Figure 2:**
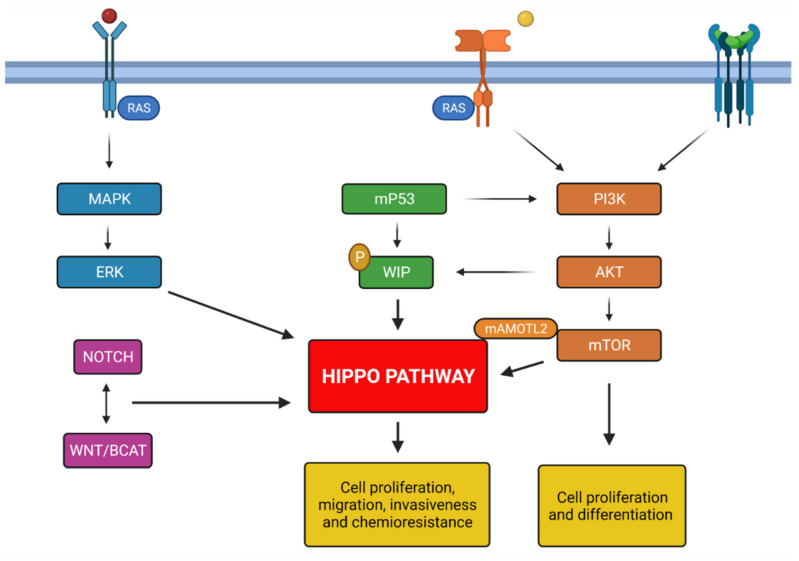
Summary of principals molecular mechanisms involved in glioblastoma genesis; all pathways described activate Hippo pathway promoting the chemoresistance and tumorigenesis. Created with Biorender.com.

The Wnt pathway contribution in GBM pathology is related to stem cell maintenance and differentiation, tumor initiation and growth, invasion potential and therapeutic resistance, thereby its dysregulation plays an important role in GBM biology [106,107]. In GBM, alterations among this pathway are more frequently found being epigenetic rather than genetic mutations in its signaling components, such as epigenetic silencing of negative Wnt regulators and overexpression of positive ones [108]. Binda et al. underlined the role of Wnt5a (a noncanonical Wnt family member) in brain invasion. The group found that the most invasive gliomas are characterized by Wnt5a overexpression associated with tumor-promoting stem-like characteristics (TPC); indeed, inhibition of Wnt5a in mesenchymal GBM TPC suppresses their infiltration capacity [109]. A cross-talk relationship exists between the Wnt pathway and other important cell signaling pathways such as Notch, Hedgehog, EGFR signaling cascades [110] and the Hippo signaling [111]. Regarding the Notch signaling, it should be underlined its high activation in GSCs (Glioma Stem Cell), where it represses differentiation and preserves stem-like properties, contributing to GBM tumorigenesis and resistance to conventional treatments [112]. Abnormal expression of many Notch components is present in brain tumors. For example, a higher expression of ASCL1, Dll1, Notch 1-3-4, and Hey1, which correlates with higher glioma grade e worse prognosis [113,114]. In addition, Notch signaling activity is reported in WHO grade IV gliomas, and can be associated with hypoxia, PI3K/AKT/mTOR and ERK/MAPK molecular pathway and finally increase malignant features of gliomas [115] (Figure 2).

## 4. Background Hippo Pathway

A fine balance between creation of new cells (proliferation) and death of extra ones (apoptosis) is vital to the correct development of organs in all multicellular organisms [116]. Malfunction of these processes contributes to cancer development. One of the main pathways which maintains homeostasis in tissues is the Hippo pathway regulating the appropriate cell number via restriction of cell growth and proliferation, and promoting apoptosis in organ growth [117,118,119]. Therefore, dysregulation of this pathway is a key maker of tumorigenesis and cancer progression [120]. The Hippo pathway’s role was first discovered in *Drosophila melanogaster* through mosaic genetic screens, whereby were identified several genes that are essential for the appropriate development of adult structures. In 1995 the first identified gene has been warts, encoding the Warts kinase (Wts) [121,122], followed by Salvador, encoding the adaptor protein Salvador (Sav) [123,124] and then, in 2003, Hippo, encoding the Hippo kinase (Hpo) [125,126,127,128]. Mutations on these genes lead to remarkable overgrowth of organ structures in flies, due to the hyperproliferative behavior of these mutated cells which are not pruned away by apoptosis, because of their resistance to it. The core of the Hippo pathway in *Drosophila* consists of these aforementioned three serine/threonine-protein kinases: Hpo, Sav and Wts. The signal kinase cascade starts with the phosphorylation of Sav by Hpo (in its active form when phosphorylated).

Their interaction leads them to a complex formation that, in turn, phosphorylates Wts and Mats (Mob as-tumor-suppressor). The Salvador kinase is called “adaptor protein”, as it brings Hpo to phosphorylate Wts. Finally, Wst-Mats phosphorylated kinase complex has as its major substrate Yorkie (Yki). As a result of this signaling cascade, Yorkie is inactivated and can not shuttle from the cytoplasm into the nucleus [129]. Homologous of the Hippo-pathway core components have been found in mammals: the mammalian Ste20-like 1 and 2 kinases (MST 1/2 in mammals and Hpo in *Drosophila*) bind the adaptor SAV1 (the WW-domain containing scaffold protein Salvador; Sav in *D.*) phosphorylating LATS1/2 (Large Tumor Suppressor homolog 1/2) and MOB1A/B (Mps One Binder 1 cofactor). LATS1/2-MOB1 phosphorylated complex (Wst-Mats complex in *D.*), in turn, phosphorylates transcriptional downstream co-activators as YAP and TAZ (Yorkie in *D.*). The kinases cascade regulates the localization of YAP and TAZ, encoded by paralogous genes. In the concrete, by phosphorylation on YAP S127 and on TAZ S89, YAP/TAZ results inactivated as it is forced to remain in the cytoplasm and bind 14-3-3 protein, which will guide YAP/TAZ to a degradation destiny [130]. This is what happens when the Hippo pathway is “on”. Conversely, when the Hippo signaling pathway is “off ”, YAP/TAZ is active as it is able to translocate into the nucleus, where it promotes the transcription of growth promoting or apoptosis inhibition genes. YAP/TAZ transcription co-activator has not DNA binding domains, but it has a TEAD-binding region (TB) and WW domains, characterized by two conserved tryptophan (W) residues. The TB makes it possible for YAP/TAZ to form complexes with TEAD1-4 (transcriptional enhancer factors), while the WW domains allow the interaction of YAP/TAZ with Runx transcription factors, which are involved in carcinogenesis and cancer metastasis [131,132].

Therefore, YAP/TAZ works as a transcription co-activator inducing the expression of target genes such as CTGF, Cyr61, MYC, PD-L1 and FGF-1 (fibroblast growth factor) [133]. Accordingly, a reduction of YAP/TAZ nuclear levels leads to down-regulation of Hippo pathway downstream gene targets. Interestingly, in response to DNA lesions the YAP WW domain interacts with p73 (a p53 family member), resulting in p73 enhanced transcription activity, that induces programmed cell death through transcription of pro-apoptotic genes [134]. Besides the Hippo kinase cascade, multiple signaling pathways and inputs could regulate YAP/TAZ, including Wnt signaling and G-protein coupled receptors (growth-factor signaling pathways), energy stress, mTOR and autophagy [135].

High levels of glucose and the activation of mevalonate pathway are some of the metabolic cues that can trigger YAP/TAZ, some others such as low glucose condition and glucagon stimulation are able to inactivate it [136,137]. YAP/TAZ can in turn influence the metabolism, allowing cell adaptation to the environment [138]. Finally, various upstream signaling mechanisms are involved in the activation of the Hippo core signaling cascade, such as molecular links with adherens junctions (AJs) and tight junctions (TJs) [139,140]. One molecular player between adherens junctions and the Hippo pathway is Neurofibromatosis type 2 (NF2 in mammals and Merlin in *D.*), an adaptor protein with the FERM-domain encoded by NF2 tumor suppressor gene. NF2 suppresses the activity of YAP in many different cells by activating the Hippo pathway [139]. Thereby, the mechanical stimuli are important for the regulation of the Hippo pathway and consequently, they influence the gene transcription induced by nuclear YAP. Moreover, the Hippo signaling is also affected by cytoskeletal remodeling due to cell junction components binding to F-actin. F-actin stress fibers are present when cultured cells grow on stiff substrates; their presence is correlated with the nuclear localization of YAP. On the contrary, when cells are cultured on soft substrate there is a cytoplasmatic localization of YAP due to lack of stress fibers. Thus, structural changes in the F-actin cytoskeleton lead to an upstream regulation of YAP localization and activity.

This mechanism is also confirmed by YAP extrusion from the nucleus in cultured cells caused by pharmacologic disruption of F-actin fibers, pointing to a key role of their contractility in regulating YAP/TAZ activity [141,142,143].

## 5. Hippo Pathway and Glioblastoma: Pharmacological Interventions

**Figure 3:**
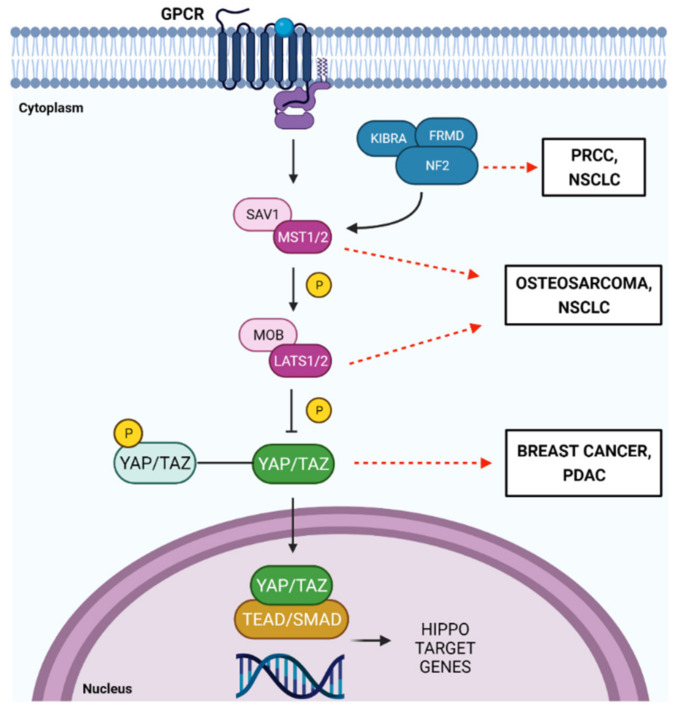
Hippo Pathway deregulated in human cancer, from the top: alteration of the tumor suppressor gene NF2 in the Papillary renal cell carcinomas (PRCC) [144]. Generally, it has been shown that the transcription factor YAP is a determining element in the progression of renal cancer, particularly in PRCC because it promotes tumor angiogenesis and its silencing increases the apoptotic rate and causes arrest of the cell cycle [146,147]. In the non-small cell lung cancer (NSCLC) the loss of function of some key Hippo Pathway genes, as well LATS1/2 and NF2, causes resistance to BET protein inhibitors (BETi) [151,152,153]. In osteosarcoma cells, methotrexate and doxorubicin (Dox) drugs damage the kinases MST1/2 and LATS1/2 activity by decreasing the phosphorylation of YAP allowing its translocation into the nucleus [148,149]. Drugs resistance is characteristic of this pathology and is one of the main causes of poor prognosis [150]. The overexpression of the YAP1 gene is present in Breast cancer (BC). YAP can induce EMT, increase the number of tumor stem cells and inhibit cell apoptosis in vitro [147,148,149]. In the Pancreatic ductal adenocarcinoma (PDAC) YAP is overexpressed in tumor samples from pancreatic cancer patients [157,158,159,160,161]. Furthermore, YAP acts as a transcriptional switch down stream of KRAS, supporting the expression of genes that promote neoplastic proliferation and stromal response [162]. Created with Biorender.com.

The Hippo pathway is an active signaling in many human cancers and participates in tumorigenic processes such as inducing EMT and stem cells, inhibition of apoptosis and promoting chemoresistance [144,145,146,147,148,149,150,151,152,153,154,155,156,157,158,159,160,161,162,163,164,165,166,167,168,169,170,171] (Figure 3).

For this reason, it can be considered a new pharmacological target, even though is still poorly investigated in brain tumors.

Gliomas include a variety of primary tumors of the CNS that develop from glial cells, such as astrocytes, oligodendrocytes, microglia and ependymal cells. Numerous signaling pathways have been studied in gliomas including Hippo pathway and in particular, its effectors YAP/TAZ, encoded by the gene WWTR1.

YAP and TAZ are crucial elements of Hippo pathway, their expression is elevated in several tumor types including gliomas [172] and correlates with the grade of malignancy, being maximal in GBM. Patients with TAZ over-expressing tumors exhibit a poor prognosis, and, in cell models, TAZ promotes tumor progression, while its knockdown prevents proliferation, tumorigenicity and invasion of glioma cells [173]. TAZ is exquisitely regulated at the level of protein stability by a wide range of stress signals such as mechanical stress, low energy status, hypoxia and osmotic stress [142,174]. These signals activate the Hippo pathway, leading to TAZ phosphorylation and subsequent cytoplasmic retention and degradation [175,176]. 

Numerous transcriptional factors as nuclear factor erythroid-derived 2-like 2 (NRF2) are involved in GBM progression. NRF2 provide a growth advantage to cancer cells in the hostile TME and promote cancer progression [177], metastasis and resistance to chemo and radiotherapy [178,179,180]. Generally, its activity is increased in GBM cell lines [181] and tumors, and elimination of NRF2 expression inhibits proliferation of GBM stem cells [182]. A recent study suggested that NRF2 might activate the Hippo pathway at TAZ level in GBM model. The authors reported that NRF2 induces the expression of WWTR1 delivering a growth, proliferative and survival signal through TAZ in GBM. It would be expected that the Hippo pathway is silent in cancer cells for TAZ to remain transcriptionally active [183].

However, among a big group of analyzed gliomas, only 4.4% exhibited mutations that might potentially inactivate the Hippo pathway [184]. Moreover, TAZ expression was increased in these tumors, therefore indicating additional mechanisms for TAZ up-regulation. So, NRF2 has been identified as one such mechanism, hence probably counteracting repressor signals and providing a tumor growth advantage, concluding that an efficient therapy for GBM must consider that high NFR2 and WWTR1 levels are predictors of chemoresistance [185]. Actually, overexpression of NRF2 and TAZ correlated with resistance to the alkylating agent TMZ, which is the gold standard treatment for gliomas [183].

According to this data, recent studies have also found that hyperactivation of YAP/TAZ is associated with resistance to canonical chemotherapies, radiotherapies, and targeted therapies [2,157,186]. Therefore, drugs targeting YAP/TAZ have been of recent interest in cancer treatment [187]. VP is a porphyrin derivative, and porphyrins related to VP cross the BBB and accumulate in the brain [188,189].

Through a phase 0 clinical trial, it has been reported that liposomal VP was effectively absorbed by GBM cells in human patients. Data showed that VP disrupted TAZ-TEAD binding and reduced YAP/TAZ protein levels and nuclear localization, confirming that VP is a dual targeting irreversible inactivator of YAP/TAZ proteins [190]. In the phase 0 study, tumor tissue from VP participants preliminarily showed low YAP/TAZ protein levels compared with a representative untreated control patient, which suggests that sufficient VP may be absorbed to disrupt YAP/TAZ protein expression in vivo in humans. These experiments have uncovered a therapeutically relevant dependency on YAP/TAZ-TEAD activity in GBM, demonstrated that these tumors display a clinically relevant therapeutic vulnerability to pharmacologic treatment with VP [85].

VPA (Valproic Acid) is an interesting candidate as a therapeutic agent for glioma’s treatment [191]. VPA is a histone deacetylase inhibitor, commonly used as anti-epileptic drug. It has been shown VPA inhibits glioma cell proliferation, migration, and invasion via the EMT process [192] and matrix metalloproteinases (MMPs) [193]. VPA is able to induce mitochondria mediated apoptosis and aptoptosis via the ERK/AKT pathway and glioma cell-cycle arrest at the G0/G1 phase [193,194].

In vitro experiments on human glioma cell line such as A172 and T98G have shown the capacity of VPA in decreasing CD44 expression, after 7 days of incubation with 1mM VPA [195]. Interestingly, CD44 (a cell-surface receptor for hyaluronan and a cancer stem cell marker) is upstream of the Hippo pathway and its depletion suppress GBM growth and sensitizes it to cytotoxic drugs in vivo [196]. CD44 is upregulated in GBM and the high level of endogenous CD44 leads to inactivation of Merlin, blocking the phosphorylation/activation of LATS 1/2, thus inhibiting its tumor suppression function.

Another way of suppressing GBM growth through the activation of the Hippo cascade, and thereby inhibiting YAP/TAZ, is by a robust pharmacological induction of SOCE (store-operated calcium entry). A pharmacologic triggering of SOCE is possible thanks to Amlodipine that enable Ca^2+^ entrance through an ORAI channel isoform in a store-dependent manner. This situation increases the cytosolic concentration of Ca^2+^ in glioma cells leading to INF2-mediated actin cytoskeleton remodeling. The new assembly of F-actin will host PKC beta II, which has been induced to translocate in the F-actin compartment by the increase of Ca2+ [197]. After the translocation, PKC beta II is activated and able to activate in turn MST 1/2 and LATS 1/2 phosphorylation.

In addition, Amlexanox (ALX), an anti-inflammatory, anti-allergic, immune-modulator drug, displays anti-glioma properties in vitro with weak adverse effects on normal cells. Incubation with ALX inhibits cellular proliferation, migration and invasion and induces G0/G1 phase arrest and apoptosis in U87MG and U251 glioma cells. Moreover, this drug directly interacts with the inhibitor of nuclear factor kappa-B kinase (IKBKE) modulating its activity and reducing its expression [198]. IKBKE bound to LATS1/2, and facilitated their polyubiquitin degradation. Meanwhile, data showed that IKBKE did not alter mRNA levels of LATS1/2 in glioma cells supporting the conclusion that this nuclear factor regulates the Hippo pathway through post-translational control of LATS1/2. According to its strong anti-proliferative activity In vitro, ALX also exhibited promising antitumor efficacy in subcutaneous glioma xenograft models. In vivo data demonstrated that ALX not only significantly reduced brain tumor growth and the expression of IKBKE but also prolonged the survival of the intracranial models, suggesting a good BBB permeability of the drug [199].

Another agent that exhibits a promising effect against glioma interacting with the Hippo pathway is Bazedoxifene (BZA). This agent is a third-generation selective estrogen receptor modulator (SERM) that shows an inhibitory effect on IL-6/GP130 in several cancers including GBM initiation and progression. Certainly, BZA acts as a GP130 inhibitor by competing with IL-6 or IL-11 for the interaction of GP130, leading to the deactivation of IL-6/GP130 signaling and delayed cancer progression. A recent study displayed that BZA treatment accelerated YAP phosphorylation, hypothesizing that there is cross-talk between IL-6/GP130 and YAP.

Therefore, deactivation of GP130 by BZA may accelerate YAP phosphorylation and degradation [200]. Silibinin, the major flavonolignan also known as silybin has been used as an antioxidant and hepatoprotective agent [201,202]. Silibinin was reported to have significant tumor suppressor functions in various cancers [203,204,205,206]. This molecule can significantly suppress the invasion and metastasis of cancer cells and metabolic activity and cell proliferation of human GBM U87MG cells [207]. Moreover, Silibinin enhances the sensitivity of various human GBM cell lines to several chemotherapeutic drugs including TMZ and etoposide. Recent data reported that this drug inhibited the phosphorylation of mTOR, p70S6K, and 4E-BP1 in human GBM cells and induced a concentration-dependent downregulation of YAP [208]. Recent studies have highlighted the anti-tumor effect of Nitidine chloride (NC) in GBM. This natural bioactive alkaloid is capable of inhibiting the malignant behavior of GBM through suppression of EMT and stem cell-like properties by modulation of the JAK2/STAT3 signaling pathway [209] and through targeting of PI3K/AKT/mTOR signaling pathway [210]. Lately, NC has been reported to have anticancer properties by activating the Hippo pathway in lungs cancer cells [211], thereby could be interesting to further investigate if NC may have a role in the activation of the Hippo signaling even in glioma cells (Table 3).

All these pieces of evidence largely support a strong involvement of the Hippo pathway in gliomas progression making this signaling pathway a promising target for the treatment of low and high grade gliomas and in particular of GBM.

## Figures and Tables

**Table 1:** Main features of glioblastoma (GBM) classification, prognosis and treatment.

GBM CHARACTERIZATION	
**Classification**	GBM is a grade IV glioma (WHO) and represents 60–70% of all gliomas. It is the most malignant and diffuse tumor of the CNS. It is common among both adults and children (males > females).
**Prognosis**	Less than 5% of patients survive more than five years after diagnosis
**Standard therapy**	Radiotherapy followed by chemotherapy with TMZ
**MDR**	Due to crosstalk between TME and GSCs Due to some deregulated signaling pathways Provoke disease recurrence, tissue tumor infiltration and metastasis

**Abbreviations:** WHO: World Health Organization; CNS: central nervous system; TMZ: Temozolomide; MDR: multidrug resistance; TME: tumor microenvironment; GSCs: glioma stem cells.

**Table 2:** GBM chemoresistance principal mechanisms.

CHEMORESISTANCE MECHANISMS	
**BBB**	Represent an obstacle to the passage of drugs Barrier endothelial cells contain large amounts of specific drug transporters called ABC superfamily (P-gp, BCRP/ABCG2 and MRP1)
**Altered DNA Repair** **DDR** **MMR**	They confer chemoresistance phenotype to GBM cells
**Aberran expression of microRNA (miRNA)**	miRNA targets are drug transporter genes, proteins involved in ABCB1/P-gp mediated chemoresistance and genes involved in DNA repair mechanisms
**Exosome release**	Induce the EMT process Activate miRNA-mediated mechanisms of gene expression regulation Promote immune escape, angiogenesis and metastasis Drugs can be internalized in exosomes and excluded from cancer cells Exosomal content may contain drug efflux pumps, fusion genes, and lncRNA

**Abbreviations**: BBB: blood brain barrier; DDR: DNA damage response; MMR: mismatch repair; EMT: epithelial–mesenchymal transition; lncRNA: long non coding RNA.

**Table 3:** Principal pharmacological therapies targeting the Hippo pathway.

PHARMACOLOGICAL OPPORTUNITIES	
**VP**	Cross BBB and accumulate in the brain Disrupt YAP-TEAD complex and decrease YAP/TAZ protein levels and nuclear localization
**VPA**	Reduce CD44 expression which is an upstream factor activating the Hippo pathway Its depletion suppress GBM growth and sensitize it to cytotoxic drugs *in vivo*
**Amlodipine**	Let an increase of cytosolic Ca2+ level which provokes an actin cytoskeleton remodeling. The new assembly of F-actin is capable to start kinase cascade and phosphorylate YAP which will degrade
**ALX**	Reduce IKBKE inhibitor expression which is bound to LATS1/2 and facilitate their polyubiquitin degradation Regulate the Hippo pathway through post-translational control of LATS1/2 Show anti-proliferative activity in vitro and also exhibit promising antitumor efficacy in subcutaneous glioma xenograft models
**BZA**	Act as GP130 inhibitor by competing with IL-6 or IL-11 for the interaction of GP130, leading to the deactivation of IL-6/GP130 signaling and accelerating YAP phosphorylation
**Silibinin**	Induce a concentration-dependent downregulation of YAP
**NC**	Increase YAP phosphorylation

**Abbreviations:** VP: Verteporfin; BBB: blood brain barrier; VPA: Valproic Acid; ALX: Amlexanox; IKBKE: inhibitor of nuclear factor kappa-B kinase; BZA: Bazedoxifene; NC: Nitidine chloride.

## Abbreviations

ALX	Amlexanox
AMOTL2	angiomotin like-2
BBB	blood brain barrier
BC	Breast Cancer
BZA	Bazedoxifene
CAR T	chimeric antigen receptor T cells modified
CNS	Central Nervous System
CSF-1	colony stimulating factor-1
CTGF	connective tissue growth factor
CTLs	cytotoxic T lymphocytes
Cyr61	cysteine Rich angiogenic inducer 61
D.	Drosophila
DDR	DNA damage response
Dox	Doxorubicin
DSBs	DNA double strand breaks
EGFR	epithelial growth factor receptor
EMT	epithelium-mesenchymal transition
FGF-1	fibroblast growth factor
GBM	Glioblastoma
Hpo	Hippo
IDO	3 dioxygenase
IKBKE	Inhibitor of nuclear factor Kappa-B Kinase
IR	Ionizing Radiation
LATS 1/2	Large Tumor Suppressor homolog 1/2
Mats	Mob As-Tumor-Suppressor
Mcp-1	monocyte chemotactic protein 1
miRNA	microRNA
MMPs	matrix metalloproteinases
MMR	mismatch repair
MOB1 A/B	Mps One Binder 1
MST 1/2	Mammalian Ste20-like 1 and 2
NC	Nitidine chloride
PRCC	Papillary Renal Cell Carcinomas
NF2	Neurofibromatosis type 2
NRF2	Nuclear Factor erythroid-derived 2-like 2
NSCLC	Non-Small Cell Lung Cancer
PD-1	programmed cell death protein-1
PDAC	Pancreatic Ductal Adenocarcinoma
PD-L1	programmed death-ligand 1
PIP3	PI-3-phosphate
RB	Retinoblastoma
Sav	Salvador
SERM	selective estrogen receptor modulator
TAMs	tumor associated macrophages
TAZ	Transcriptional Co-activator with PDZ-binding motif
TB	TEAD-Binding
TGF-b	Transforming Growth Factor beta
TMZ	Temozolomide
TPC	tumor promoting stem-like Characteristics
VPA	Valproic Acid
VP	Verteporfin
Wts	Warts
YAP	Yes-Associated Protein
Yki	Yorkie
